# The Institutional Library

**Published:** 1920-08-07

**Authors:** 


### THE INSTITUTIONAL LIBRARY.

**Notes on a Cellar-Book.** By George Saintsbury. (London : Macmillan and Co. 1920. Price 7s. 6d. net.)

It is difficult to praise with due restraint this learned and delightful book, which if (to quote a phrase used by the author) "the general common sense of the country" had not "been weakened by a washy overflow of so-called education," could stem the tide of ignorance and prejudice on the subject of wine. Professor Saintsbury reminds his readers that "there is absolutely no scientific proof of a trustworthy kind that a moderate consumption of sound alcoholic liquor does a healthy body any harm at all," while all experience and tradition are in its favour. The Hospital once published an elaborate report on beer to prove its value, and Mr. C. Louis Leipoldt, F.R.C.S., when a member of the staff, wrote a delightful volume called "Common-sense Dietetics," which contained not only an exposure of the modern fads, but some admirable chapters on the art of dining, which he had studied with discrimination in many parts of the world. After reminding his readers that "the alleged costliness of alcoholic drinks is entirely due to legislation," a point which their opponents carefully omit, Professor Saintsbury discusses wines and their vintages, spirits, liqueurs, mixed drinks, beer and cider. Each chapter is the result of ripe experience, and treats an important subject with the care which it deserves. A cellar used to be among the scholar's pleasures, and this cellar-book is full of information on each wine, its storage, and the kind of glass in which it should be drunk. The menus at the end make excellent reading. His mode of establishing a cask of whisky is approved by tradition, and we can endorse his preference for cidercup, for which he considerately supplies a personal recipe. Mr. Saintsbury's taste is catholic, and he puts in a good word for vodka, but it is a sad sign of the times that the proper way to drink absinthe should require description, and that he should have to affirm that draught beer is preferable to bottled. The book abounds in good stories, including one of "a physician of distinction who had made himself rather conspicuous by frequent tirades in print against alcohol," but who "positively disturbed his neighbours at a public dinner by outcries against the corking of some, as it seemed to me, quite innocent champagne." Another case of a man whose practice was better than his theory was that of a teetotaller who offered us on one occasion some excellent Steinburg cabinet. There is some good-humoured chaff at the "toxic euphoria" alleged by teetotallers to follow on a glass of wine, and words are now used so loosely in these controversies that Mr. Saintsbury might have added that fatigue is now plausibly said to be a form of "intoxication," were not opponents of this class beyond the reach of scholarly argument. The book is an essay in common sense, and its robust and refreshing tone sends the reader on his way rejoicing.

**Animal Experiments and Surgery.** By Walter G. Spencer, M.S., F.R.C.S. (University of London Press, Ltd. Price 6s. net.)

Verbal conflicts on this increasingly vital problem in medical research have for some time past been brought with varying prominence before the general public. Whether this has occurred on a critical occasion, such as the recent defeat of the Dogs Bill, or through protracted arguments carried on in some journalistic correspondence column, there has always been something of a haze spread before the lay spectator's eyes. It is a result probably quite unintentional on the part of either of the conflicting parties; the scientific case is not only impregnable but absolutely convincing to the least educated in such matters.

Therefore we can most heartily welcome the appearance of a new work by Mr. Walter Spencer, M.S., F.R.C.S., which was recently published by the University of London Press. "Animal Experiments and Surgery" suggests at first sight considerable limitation of the particular fields described, but actually this book has a very wide scope. Essentially it contains the Hunterian Lectures delivered under the same title, which directly owe their origin to the Dogs Bill itself. When delivered these lectures temporarily accomplished the task they set out to achieve, but we doubt whether they received that public attention which was really necessary. Technically correct and intensely interesting to the surgeon, young or old, the great point we would urge upon the reading public is that Mr. Spencer's book does not contain just hard scientific fact. It is indeed a small volume which anyone might find a more than attractive companion on the average railway journey.

**Child Welfare.** By J. Sim Wallace, M.D., L.D.S. Baillière, Tindall & Cox. 1919. Pp. 102. Price 5s. net.)

Dr Wallace has been for years one of the most insistent of the dentists and physicians who have been dinning into the ears of the British public the importance of cleaning their teeth, or of "oral hygience," as it is technically termed. So far the success that has attended their efforts has been but moderate, but there are signs that things are on the mend. If the British people have not yet awakened to the importance from the public health point of view of keeping their mouths reasonably clean and free from caries, at least a few of our governing officials and public bodies are beginning to realise the truth. Only the other day, it was pleasing to see, the *Times* admitted (not in the medical correspondent's column) that the British people scandalously neglect their teeth to their own great detriment; so the leaven is beginning to work, even if slowly. To judge by what one sees in practice, the middle classes are now realising the truth of what the dentists have been telling them; but the working classes have not yet mended their ways, and much educational work is necessary to get them to do so. This can only be done with any prospect of success by catching them young, *i.e.*, at school. Dr. Wallace's book consists of a series of addresses and papers on this most important topic, delivered at various times and places. They are somewhat provocative in tone but quite admirable in substance.

**Ward Tales.** By E. Chivers Davies. (John Lane. 1920. Pp. 211. Price 5s. net.)

An amusing series of sketches of work in a V.A.D. hospital, by an observant writer with a sense of humour, whose work will be acceptable to anyone who at this hour wishes to revive memories of hospital days during the war. But the regular institutional worker will agree with the nurse in the first chapter on the subject of distended pig-tubs and wholesale smashing: "It doesn't appear to me to be anything in the *least* funny."

**The Institutional Library**—(*continued*).

**The Science of Eating.** By Alfred W. McCann. (Evans Bros. Pp. 408. Price 10s. 6d.)

This work is an attempt to expose what the author of it regards as a huge series of criminal frauds on the citizens of the United States, committed by the big commercial interests which manage the wholesale food supplies of that country. He believes that they are—many, if not most of them—engaged in selling rotten, disease-producing foodstuffs at profiteering prices, regardless of the consequences to the public and seriously to the detriment of the national health. It is quite possible that many of his contentions are true; and by no means unlikely that many of his strictures apply, *mutatis mutandis*, to conditions in this country also. But his whole work is so hysterical in manner, so bombastical and egotistical, that the book is little likely to "go down" with readers on this side of the Atlantic, and so to produce the benefit which the author doubtless hopes and intends it to confer upon us.

**Transactions of the Incorporated Sanitary Association of Scotland, 1919.** (Glasgow: 83 Bath Street.)

We find in these annual Transactions the features usual of late years: a sturdy nationalism—their thistle is unmistakeably Scotch, and not of "the English or donkey kind"—a view of the war, which (like that, unfortunately, of so many people) is not *bien documenté*, and a dozen or so of very useful papers on sanitary subjects. "Ex-Bailie" Russell brought out well the contradictory opinions existing as to the etiology of high infantile mortality; the most frequent cause being variously stated as syphilis, lack of sanitary administration, and ignorance of the mother, by Mrs. Scharlieb, Dr. Newsholme, and Sir George Newman respectively. The layman has certainly scored a point here. Dr. Dewar has a witty and iconoclastic article on the use of disinfectants, which, like most iconoclastic deliverances, evoked a good deal of discussion. A speaker on the milk question drew attention to a discouraging phenomenon, viz. the sale and export to foreign countries of tubercle-free cattle, with no import of the same, so that the infected ones are left in greater number at home to breed from. And so one might go on. Any sanitarian, professional or lay, will find this a very interesting and compact brochure.

**All About Baby.** By A. R. Neuman. (London: Constable. Price 1s. 6d. net.)

This is a re-issue of a little book published last year. Occasional correction is needed, as in the statement that glycerine cannot now be bought. The sixteen chapters are simply written, and convey wise advice on feeding, clothing, training, and so forth of an infant which the nurseless mother can well follow. The avoidance of midnight feeding is advocated, and the sections on childish ailments wisely note the points at which the doctor should be called in. Attention to different kinds of crying and their indications is a detail of management not always found in these books. Altogether this may be recommended as an inexpensive and useful gift to a young mother of fair education.

**The Wife and Mother.** By Albert Westland, M.D. (C. Griffin and Co. 7th edition. 1920. Pp. 282. Price 5s.)

This long-established book of advice for young wives and mothers—it was first published in 1892—has now reached a seventh edition, which is sufficient evidence that it is well conceived and popular. It is commendably free from the vein of mawkish sentimentality by which so many books of this type are characterised; and is reliable and accurate. For many readers it may be a shade too technical—the author seems to expect a higher standard of educational proficiency from those he writes for than most of them are likely to evince. This, and a distinct shirking of sexual issues, are the only real criticisms of an excellent book of its kind.

**A Handbook of British Mosquitoes.** By William Dickson Lang, M.A., Sc.D. Printed by order of the Trustees of the British Museum, and sold by Longmans Green & Co., Paternoster Row, E.C. 4, and at the British Museum (Natural History), Cromwell Road, S.W. 7.

Conforming to the usual high standard of work put out in these Museum publications, the work on mosquitoes is invaluable to those interested in the disease and the possibilities of its spread in this country. It has, besides its great entomological utility, a distinct bearing upon one of the great medical questions of the day.

**Sanitation for Public Health Nurses.** By Hibbert Winslow Hill, M.D., D.P.H., etc. (New York: MacMillan Co. 1919.)

Sanitation is presumably one of the words which have a somewhat different meaning in America to that usually understood in this country. Dr. Hibbert Winslow Hill deals very little in this book with drainage, ventilation, or other kindred subjects; indeed, one of his main contentions is that cleanliness, and the avoidance of "bad smells"—though desirable enough for æsthetic reasons—have little or nothing to do with the maintenance of health or the elimination of disease.

The first two-thirds of the book consist of an excellent bacteriological survey of all varieties of epidemic diseases, from leprosy to common cold, and should be most useful in assisting the health nurse to recognise any case that may come to her notice, and take really effective means to prevent spread of the infection. The remaining chapters deal with the main channels by which infection may be transmitted other than by direct contact—food, milk, water, flies, etc.—including a very clear exposition of the dangers arising from the feeding of infants otherwise than in Nature's way.

The last chapter deals with the use of statistics, with suggestions for interesting the community in infant welfare work; but it is doubtful whether it is quite necessary to explain the law of averages in such detail to readers who have sufficient education to master the earlier part of the book.

**Venereal Disease: Its Prevention, Symptoms, and Treatment.** By H. Wansey Bayly. (London: J. & A. Churchill, 1920. Pp. 152. Price 10s. 6d. net.)

In this book Mr. Hugh Wansey Bayly tries to compress the whole subject of venereal disease into some 150 pages, a matter of some difficulty. It must be confessed, however, that he has succeeded as well as could be expected. The orthodox treatment of syphilis is well dealt with; some useful notes on the value of the Wasserman reaction are given, and some practical hints on running a clinic.

As to gonorrhœa there is much of value in the book, enough to send the reader to larger and more detailed works, and to drive home the fact that the treatment of gonorrhœa and its complications is an art calling for careful study and infinite patience. The original illustrations are striking, but whether they would convey much information to any person unacquainted with the conditions portrayed is questionable.

